# Low doses of cholera toxin and its mediator cAMP induce CTLA-2 secretion by dendritic cells to enhance regulatory T cell conversion

**DOI:** 10.1371/journal.pone.0178114

**Published:** 2017-07-31

**Authors:** Cinthia Silva-Vilches, Katrien Pletinckx, Miriam Lohnert, Vladimir Pavlovic, Diyaaeldin Ashour, Vini John, Emilia Vendelova, Susanne Kneitz, Jie Zhou, Rena Chen, Thomas Reinheckel, Thomas D. Mueller, Jochen Bodem, Manfred B. Lutz

**Affiliations:** 1 Institute of Virology and Immunobiology, University of Wuerzburg, Wuerzburg, Germany; 2 Department of Physiological Chemistry, University of Wuerzburg, Wuerzburg, Germany; 3 BioLegend Inc., San Diego, California, United States of America; 4 Institute of Molecular Medicine and Cell Research, and BIOSS Centre for Biological Signaling Studies, University of Freiburg, Freiburg, Germany; 5 Department of Molecular Plant Physiology and Biophysics, University of Wuerzburg, Wuerzburg, Germany; Purdue University, UNITED STATES

## Abstract

Immature or semi-mature dendritic cells (DCs) represent tolerogenic maturation stages that can convert naive T cells into Foxp3^+^ induced regulatory T cells (iTreg). Here we found that murine bone marrow-derived DCs (BM-DCs) treated with cholera toxin (CT) matured by up-regulating MHC-II and costimulatory molecules using either high or low doses of CT (CT^hi^, CT^lo^) or with cAMP, a known mediator CT signals. However, all three conditions also induced mRNA of both isoforms of the tolerogenic molecule cytotoxic T lymphocyte antigen 2 (CTLA-2α and CTLA-2β). Only DCs matured under CT^hi^ conditions secreted IL-1β, IL-6 and IL-23 leading to the instruction of Th17 cell polarization. In contrast, CT^lo^- or cAMP-DCs resembled semi-mature DCs and enhanced TGF-β-dependent Foxp3^+^ iTreg conversion. iTreg conversion could be reduced using siRNA blocking of CTLA-2 and reversely, addition of recombinant CTLA-2α increased iTreg conversion *in vitro*. Injection of CT^lo^- or cAMP-DCs exerted MOG peptide-specific protective effects in experimental autoimmune encephalomyelitis (EAE) by inducing Foxp3^+^ Tregs and reducing Th17 responses. Together, we identified CTLA-2 production by DCs as a novel tolerogenic mediator of TGF-β-mediated iTreg induction *in vitro* and *in vivo*. The CT-induced and cAMP-mediated up-regulation of CTLA-2 also may point to a novel immune evasion mechanism of *Vibrio cholerae*.

## Introduction

It is well accepted that DCs play a central role in the induction of adaptive immune responses as well as in the maintenance of peripheral tolerance [1,2]. DCs are able to sense a wide array of pathogens and mount an appropriate T helper cell response through the expression of pattern-recognition receptors such as Toll-like receptors [3]. Naive CD4^+^ T cells can differentiate into a variety of CD4^+^ T cell subsets characterized by the cytokine produced: Th1 cells secrete predominately IFN-γ, Th2 cells release IL-4, IL-5 and IL-13 and Th17 cells typically produce IL-17 [4]. Although the contribution of DCs for CD4^+^ T cell polarization is under debate [5,6] several DC-derived mechanisms have been described to significantly direct Th cell phenotypes. Depending on the stimulus DCs change their maturation status by up-regulating surface expression of MHC-II and co-stimulatory molecules and by producing a defined set of cytokines to optimally induce distinct Th cell responses [7–9].

Adaptive immune responses are also characterized by the activation and differentiation of CD4^+^ regulatory T cells (Tregs). They consist of pre-existing thymus-derived, natural Tregs (nTregs) or are induced from naive CD4^+^ T cells in the periphery (iTregs). Both allow effector cell responses against pathogens but help to terminate these responses at later time points and prevent auto-reactive T cells from autoimmune attack [10–12]. While effector CD4^+^ T cell responses are characterized by their subsequent polarization into Th1, Th2, Th9 or Th17 subsets, fulfilling specific tasks in defending from viruses, intracellular and extracellular bacteria or fungi, the transcriptional regulation of the differentiation of Tregs from naive T cells or during polarized effector Th1 or Th2 responses for counter-regulation is still less well understood [13–15]. The induction of effector and regulatory T cells has been followed also for the balance between Th17 and Foxp3^+^ iTregs [16]. In this setting IL-6 plays a pivotal role in determining whether in the presence of TGF-β alone Foxp3^+^ iTregs will develop or in the combined activity of TGF-β plus IL-6 a Th17 response will be raised. Although DCs essentially influence Th17 cell polarization or iTreg induction, most of these studies did not address the DC subset, DC maturation state or by which molecular mechanisms DCs can act in a tolerogenic way [15].

Our previous work indicated how LPS- or TNF-matured DCs could induce polarized Th1 or Th2 CD4^+^ T cell responses, respectively [17]. In this context we also tested CT since it had been reported as an adjuvant that favored Th2 immunity [18–20]. However, also the induction of Th17 responses [21,22] or regulatory Foxp3^-^ IL-10^+^ Treg cells [23] but not Th1 effector cells [24] have been described for CT. *In vivo* experiments indicated that the route of application and the use of CT versus its subunit B alone (CTB) could decisively direct immunogenicity or tolerogenicity of model antigens conjugated to CT or CTB. The oral route of CTB administration appears as dominantly tolerogenic showing induction of Foxp3^+^ and Foxp3^-^ Tregs in mice [25]. Consequently, CTB-peptide conjugates have been tested in studies with patients suffering from Behcet's diesease and demonstrated substantial benefit [26]. However, the molecular mechanisms how CT or CTB induce Tregs are not known.

While immature DCs have been found to act as tolerogenic DCs via induction of T cell anergy or regulatory T cells [27], we found in addition that DCs matured with TNF reached only a semi-mature state and resembled tolerogenic steady state migratory DCs [28,29]. This enables them convert MOG peptide-specific naive T cells into IL-10-producing Foxp3^-^ Tr1 cells in the EAE model [30]. In contrast, DCs reaching a fully mature state after LPS treatment resulting in strong inflammatory cytokine production and inducing Th1 immunity enhanced EAE symptoms [17,30]. The phenomenon of semi-maturation was later confirmed by global transcriptional analyses and showed that TNF-DCs initially instructed a Th2-like response that was converted into a Tr1 response after repeated injections [17].

Here we show that high doses of cholera toxin (CT^hi^) do not induce TNF-like semi-maturation nor LPS-like DC full maturation, but a CT-specific gene expression profile of full maturation promoting Th17 immunity. In contrast, low doses of CT (CT^lo^) or its known intracellular mediator cAMP induced a semi-mature DC state, but distinct from TNF. CT^hi^-, CT^lo^- or cAMP-mediated DC maturation were unique by induction of the tolerogenic molecule CTLA-2.

The CTLA-2 protein was first identified in murine activated cytotoxic T cells and mast cells [31]. The mouse carries two protein isoforms CTLA-2α and -2β, which are 90% identical [32] and have been described as inhibitors of the endolysosomal cysteine protease cathepsin L [33,34]. However, also a cathepsin L-independent function of CTLA-2 has been reported [35]. CTLA-2β seems to play an important role during embryogenesis since the respective homozygote gene-deficient mice show complete pre-weaning lethality (http://www.mousephenotype.org/data/genes/MGI:88555). In adult animals CTLA-2 mRNA expression was found preferentially in cells of immune-privileged organs such as murine brain cells [36], uterus [37], placenta [38], retinal pigment epithelial cells [39] and corneal endothelial cells [40]. It appears that CTLA-2α contributes to the immune privilege of the eye by promoting activation of TGF-β and thus, supporting local Foxp3^+^ iTreg generation [39,41]. Hence, we hypothesized that CTLA-2 expression in DCs might serve tolerogenic functions.

Here we established that CT and cAMP induce CTLA-2 in DCs. Subsequently, CTLA-2 acts as a tolerogenic molecule by promoting TGF-β dependent Foxp3^+^ Treg generation *in vitro*. Furthermore, CT^lo^- and cAMP-treated DCs, which are unable to secrete Th17-polarizing cytokines, induce *in vivo* tolerance in the EAE model. Our data further suggest a CTLA-2-specific immune evasion mechanism by *Vibrio cholerae*. This mechanism may be further exploited to study CTLA-2-mediated immunosuppression in other autoimmune models, allergies or transplantation.

## Results

### Cholera toxin- and cAMP-matured DCs express CTLA-2α and β

**Fig 1:**
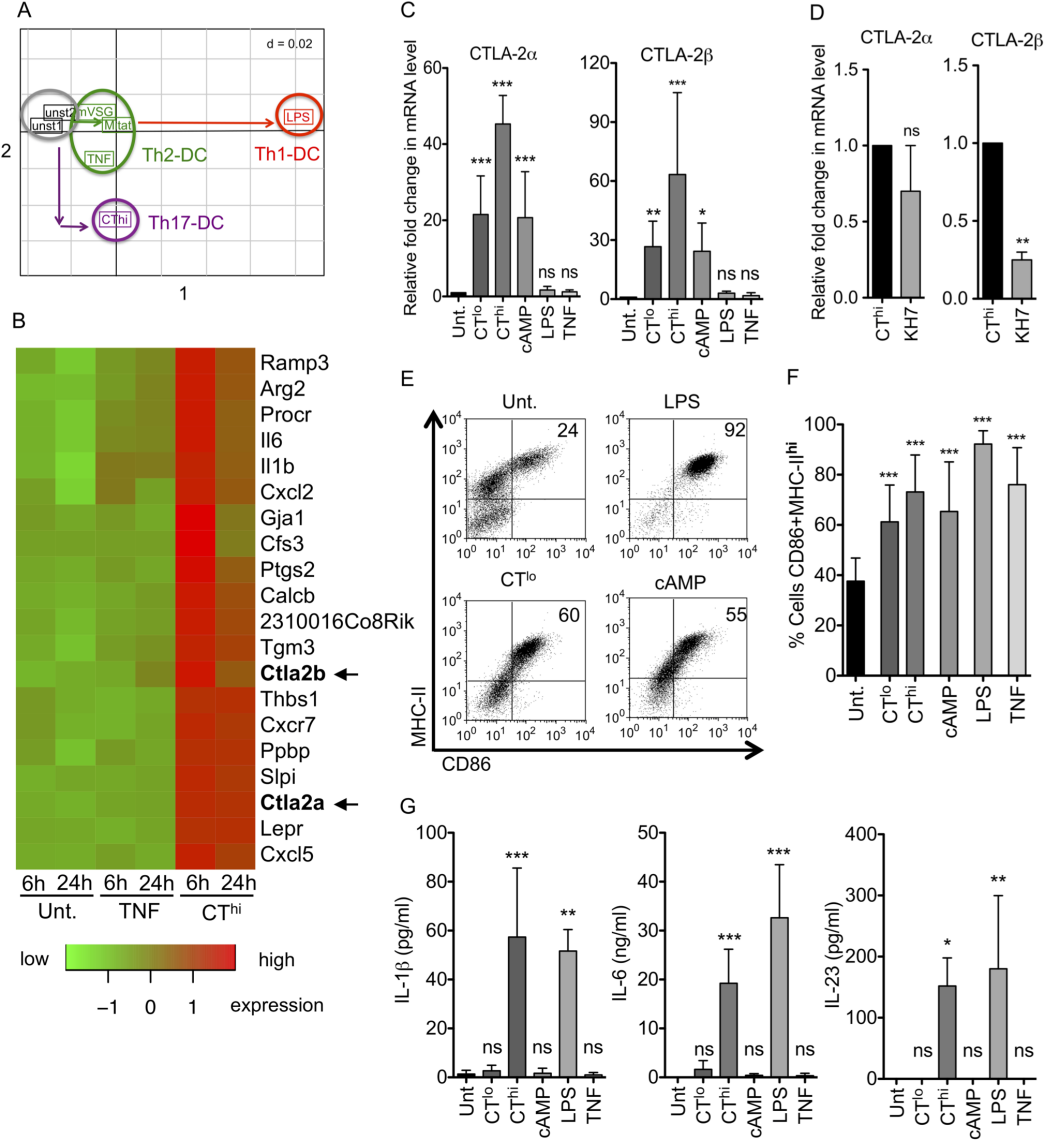
CT and cAMP induces both CTLA-2α and -2β during DC maturation. BM-DCs were differentially matured with the indicated stimuli for 16h unless otherwise specified. (A) Principal component analysis comparing untreated BM-DCs, DCs matured for 6h with the Th2-inducing stimuli TNF, the *Trypanosoma brucei* antigens Mitat or mVSG, and the Th1-inducing LPS from our previous study [17] with CT^hi^ (1 μg/ml) matured Th17-inducing DCs investigated in this study. (B) Heat map displaying the top 25 genes regulated in DCs after CT^hi^ or TNF stimulation compared with untreated DCs. Data show single time point values of a single microarray. (C) Densitometric analysis of CTLA-2α and -2β expression levels determined by RT-sqPCR normalized to β-actin and relative to untreated control for n = 4 experiments; CT^lo^ (0.1 μg/ml). (D) CT^hi^ stimulation of BM-DCs ± the cAMP inhibitor KH7 before RT-sqPCR normalized to β-actin of n = 3 independent and pooled experiments. (E) DC maturation analysis by flow cytometry of CD86 and MHC-II on CD11c positive cells. (F) Statistical evaluation of D with respect to untreated control for n = 5 experiments. (G) IL-1β, IL-6 and IL-23 secretion by differentially stimulated DCs measured by ELISA n = 3 experiments. Statistical analysis was performed with respect to untreated control. Error bars represent mean ± SD. One Way ANOVA, Dunnett post-test. *p<0.05, **p<0.01, ***p<0.001, ns: not significant. For D, student's t test, n = 3, *p<0.05.

Previous work in our laboratory intended to identify gene expression profiles of LPS- or TNF-matured bone marrow-derived DCs (BM-DCs) for induction of Th1 or Th2 responses, respectively [17]. Here we compared the effects of CT on the gene expression profiles of murine BM-DCs with the previously identified profile. A principal component analysis confirmed that Th1- and Th2-inducing DCs show a gene signature clearly different from DCs treated with high doses of 1 μg/ml CT (termed CT^hi^) (Fig 1A) and potentially indicating a Th17-inducing gene signature as suggested by others [42]. Among the top 25 genes regulated after 6 or 24h of CT^hi^ stimulation CTLA-2α and CTLA-2β were specifically up-regulated which was not observed with untreated or TNF matured DCs (Fig 1B) or by LPS or Trypanosoma-derived variant-specific surface glycoproteins (VSGs) stimulation [17]. Thus, CTLA-2α and CTLA-2β induction by CT in DCs is unique for CT and has not been reported before.

Next we tested whether the induction of CTLA-2 by CT was dose-dependent and which down-stream mediator would account for the CT effect. CT is internalized by the cells via the GM1 ganglioside and once inside the cells, subunit A of the CT complex leads to activation of adenylate cyclase and an ensuing elevation in cyclic AMP (cAMP) levels, which triggers a wide spectrum of intracellular signaling events [43]. Therefore, we used a cell membrane-permeable analog of cAMP, 8-CPT-cAMP (referred from now on simply as cAMP). The cAMP concentration of 100μM correlated with 0.1μg/ml CT for CTLA-2α and CTLA-2β induction and DC surface marker expression as revealed by dose titration experiments (not shown). The specific upregulation of both CTLA-2α and CTLA-2β mRNA was observed independent of using different doses of CT such as high doses of 1 μg/ml (CT^hi^) or low doses of 0.1 μg/ml (CT^lo^) or by its intracellular mediator cAMP, but not other stimuli could be confirmed by sqPCR (Fig 1C). To test whether CTLA-2α/β induction by CT required cAMP, the adenylate cyclase inhibitor KH7 was added during CT^hi^ stimulation of BM-DCs. While CTLA-2α showed only a trend of reduced mRNA levels, the reduction of CTLA-2β was significant (Fig 1D), indicating that cAMP is indeed the signaling intermediate of CT, at least for CTLA-2β induction. While CT^hi^ and CT^lo^ as well as cAMP treatments of BM-DCs induced the expression of surface maturation markers (Fig 1D and 1E), only CT^hi^-DCs were found to secrete IL-1β, IL-6 and IL-23 (Fig 1G). As expected, LPS induced both surface markers and cytokines similar to CT^hi^ (Fig 1F and 1G). TNF treatment led to a semi-mature phenotype, up-regulating maturation markers but lacking cytokine production (Fig 1F and 1G), similar to the treatment under CT^lo^ or cAMP conditions. Thus, the gene expression profile of CT^hi^-DCs predicts the induction of Th17 cells and is different from Th1- and Th2-inducing DCs. All three conditions of CT^hi^-DCs, CT^lo^-DCs or cAMP-DCs induced CTLA-2α and CTLA-2β mRNA that may predict a contribution to Treg induction.

### CT^hi^-DCs favor Th17 responses while CT^lo^- or cAMP-DCs induce CTLA-2-dependent Foxp3^+^ iTreg conversion *in vitro*

**Fig 2:**
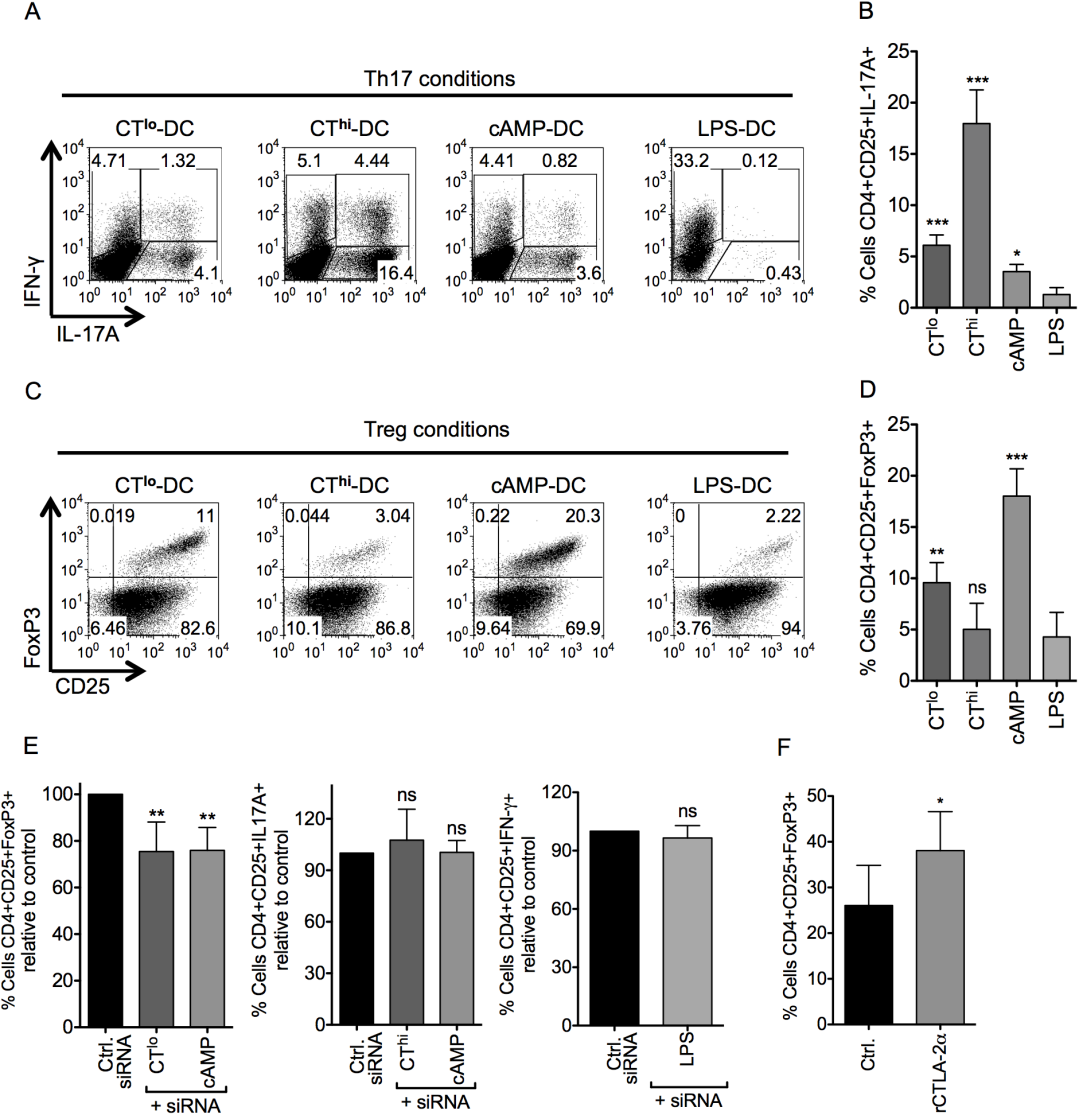
CT^lo^- or cAMP-DCs enhance TGF-β dependent Foxp3^+^ iTreg conversion via CTLA-2 *in vitro*. OT-II T cells were co-cultured with C57BL/6 BM-DCs matured with the indicated stimuli (CT^lo^ 0.1 μg/ml; CT^hi^ 1 μg/ml; cAMP 100 μM; LPS 0.1 μg/ml) in the presence of (100 ng/ml) OVA_327-339_ peptide and TGF-β (2 ng/ml) + IL-6 (20 ng/ml) for Th17 polarizing conditions or TGF-β (2 ng/ml) for iTreg conversion conditions. (A) After 5 days cells were re-stimulated for 6 h with 30 ng/ml PMA + 1 μg/ml ionomycin in the presence of 5 μg/ml brefeldin A + 2μM monensin and IL-17A and IFN-γ producing OT-II cells were analyzed by flow cytometry in a CD4^+^Vβ5^+^ gate. (B) Statistics of A with respect to LPS-DCs used as negative control for n = 5 experiments. (C) After 5 days cells were analyzed by flow cytometry for Foxp3 and CD25 expression in a CD4^+^Vβ5^+^ gate. (D) Statistics of C with respect to LPS-DCs used as negative control for n = 5 experiments. (E) OT-II naïve T cells were co-cultured with BM-DCs first transfected with 5 μM siRNA against CTLA-2, scrambled siRNA or mock-electroporated (pooled controls) and then, treated with different stimuli. Graphs represent changes in the frequency of iTreg (n = 7), Th17 (n = 6) and Th1 (n = 3) normalized to control. Numbers inside each dot plot represent the cell frequency in each quadrant. Error bars represent mean ± SD. One Way ANOVA, Dunnett post-test. *p<0.05, **p<0.01, ***p<0.001, ns: not significant. (F) C57BL/6 naïve CD4^+^CD25^-^ T cells were cultured in the presence of TGF-β plus recombinant CTLA-2α. After 3 days cells were analyzed by flow cytometry for Foxp3 and CD25 expression in a CD4^+^ gate (n = 6). Error bars represent mean ± SD. Two-tailed paired Student’s t test. *p<0.05.

After having analyzed the maturation profile of CT- or cAMP-matured DCs, we wanted to determine the type of CD4^+^ T helper/Treg cell response that would be induced by these differentially matured DCs. CT- or cAMP-treated DCs were co-cultured with OT-II T cells in the presence of Th17-polarizing cytokines (TGF-β + IL-6) or under Treg conversion conditions (TGF-β). CT^hi^-DCs clearly induced a Th17 profile in polarization assays while CT^lo^- and cAMP-DCs showed a much lower Th17 conversion (Fig 2A and 2B). LPS-DCs induced Th1 cells even under Th17 polarizing conditions, indicating that IL-12 production may overrule the Th17 conditions (Fig 2A and 2B). Under Treg conversion conditions LPS- and CT^hi^-matured BMDC were unable to generate CD4^+^CD25^+^Foxp3^+^ T cells (iTreg), while CT^lo^ or cAMP-DCs clearly favored Foxp3 expression (Fig 2C and 2D). Of note, the generation of Foxp3^+^ Tregs was strictly dependent on the addition of exogenous TGF-β (not shown). Although CT^hi^-DCs induced the expression of CTLA-2α/β similar to cAMP-DCs, the observed results indicate that the Th17 polarizing effects by IL-1β, IL-6 and IL-23 [44] dominate over the CTLA-2-mediated effects on Treg conversion.

To assess if these observations were indeed prompted by CTLA-2, we performed *in vitro* T cell polarizations assays blocking CTLA-2α/β in DCs by siRNA. The results indicate that inhibition of CTLA-2α/β in both CT^lo^- and -cAMP-matured DCs led to an impaired capacity to convert naïve T cells into iTregs, but had no effects on Th17 or Th1 polarization (Fig 2E). Likewise, addition of recombinant CTLA-2α into an anti-CD3 antibody mediated-Treg assay significantly increased the frequency of iTregs in the presence of TGF-β (Fig 2F).

Taken together, these results suggest that CT^lo^- and -cAMP-matured DCs enhance the TGF-β dependent conversion of naïve T cells toward iTreg supported by CTLA-2α/β. Under CT^hi^ conditions the effect of CTLA-2α/β seems to be outcompeted by the production of Th17 polarizing cytokines IL-1β, IL-6 and IL-23 by the DCs.

### Cathepsin L expression is not required in CT/cAMP-DCs for iTreg conversion

**Fig 3:**
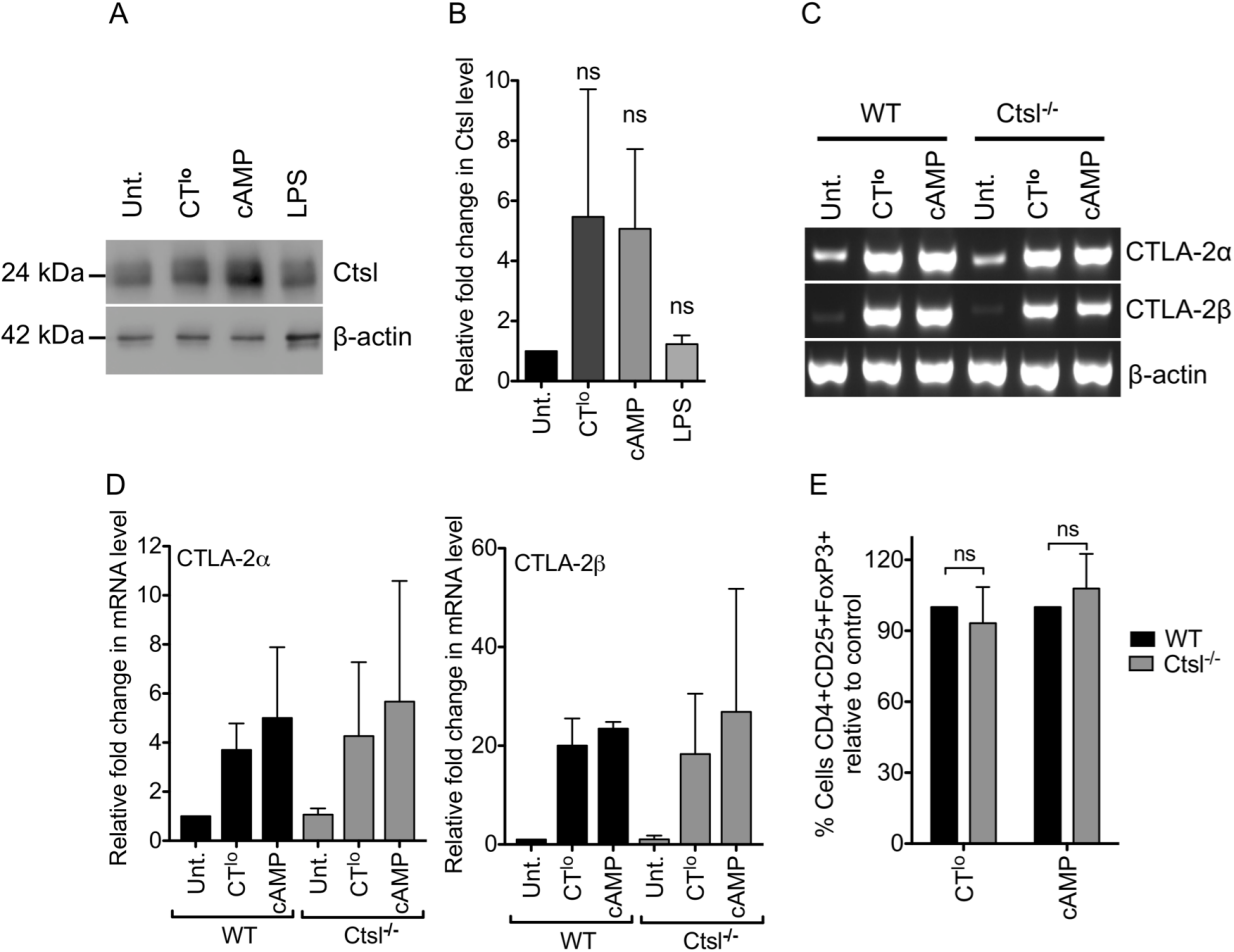
Cathepsin L expression in CT/cAMP-DCs does not influence iTreg conversion. (A) BM-DCs were differentially matured with the indicated stimuli for 10 h (CT^lo^ 0.1 μg/ml; cAMP 100 μM; LPS 0.1 μg/ml). Total cell lysates were analyzed for mature Ctsl protein content by Western Blot. One representative experiment is shown. (B) Densitometric analysis of Western blot data of 3 mice per group from 3 independent experiments performed like the one shown in A, normalized to β-actin and relative to untreated control. (C) BM-DCs were stimulated with CT^lo^ or cAMP for 4 h. CTLA-2α and -2β mRNA expression was determined by RT-sqPCR, representative experiment, (D) Densitometric analysis of mRNA data of 3 mice per group from 3 independent experiments performed like the one shown in C, normalized to β-actin and relative to WT untreated control. (E) OT-II T cells were co-cultured with WT or *Ctsl*^*-/-*^ BM-DCs-treated with CT^lo^ or cAMP for 4 h in the presence of 100 ng/ml OVA_327-339_ peptide and 2 ng/ml TGF-β. After 5 days cells were analyzed by flow cytometry for Foxp3 and CD25 expression in a CD4^+^Vβ5^+^ gate. Data represent the change in the frequency of iTreg conversion using *Ctsl*^*-/-*^ DCs normalized to WT DCs of n = 5 experiments. Error bars represent mean ± SD. (B) One Way ANOVA, Dunnett post-test. (E) Two-tailed Student’s t test. ns: not significant.

Since both CTLA-2α/β molecules have been described to inhibit cathepsin L (ctsl) [33,34] we tested whether deficiency of cathepsin L in CT-DCs would influence the CTLA-2-mediated Treg conversion. Western blot analysis revealed that cathepsin L, the substrate of CTLA-2α/β, was induced to a similar extent when DCs were treated with CT or cAMP but not using LPS (Fig 3A and 3B), indicating that substrate and inhibitor are induced under the same conditions. However, DCs derived from *Ctsl*^-/-^ mice stimulated with CT and cAMP could induce the expression of CTLA-2α and -2β mRNA to the same level as WT DCs (Fig 3C and 3D). When WT or *Ctsl*^-/-^ DCs treated with CT^lo^ or cAMP were used in a Treg conversion assay, they were similarly able to generate iTregs (Fig 3E). This indicates that cathepsin L expression in CT/cAMP-treated DCs does not influence iTreg conversion and also suggests that CTLA-2α/β might promote iTreg conversion independently of its interaction with DC-derived cathepsin L.

### Both CT^lo^ and cAMP-treated DCs are protective in EAE

**Fig 4:**
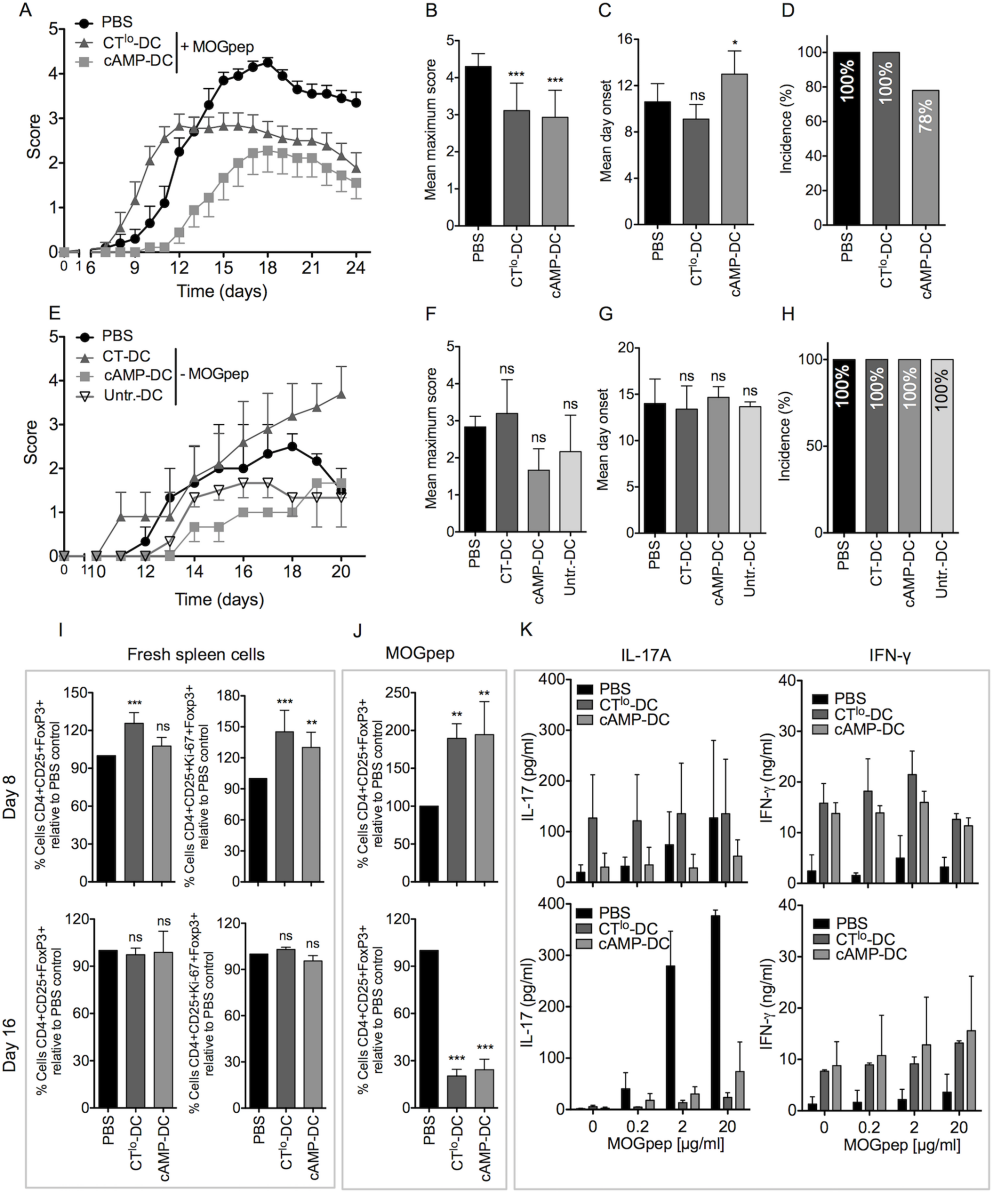
Both CT^lo^ and cAMP-matured DCs are protective in EAE. (A) CT^lo^ (0.1 μg/ml) or cAMP (100 μM) treated BM-DCs loaded with 40 μg/ml MOG_35-55_ peptide were injected *i*.*v*. at days -7, -5, -3 (2×10^6^/mouse) before MOG-specific EAE induction at d0. Control mice received PBS injections. Average disease scores were monitored for a total of 10 mice per group from two independent experiments. Error bars represent mean ± SEM. (B), (C) and (D) Statistical analyses of different other clinical parameters derived from A. Error bars represent mean ± SD of pooled results from n = 10 mice. (E) CT^lo^ (0.1 μg/ml), cAMP (100 μM) treated BM-DCs or untreated BM-DCs were injected *i*.*v*. at days -7, -5, -3 (2×10^6^/mouse) before MOG-specific EAE induction at d0. Control mice received PBS injections. Average disease scores were monitored for a total of 6 mice per group of two independent experiments. Error bars represent mean ± SEM. (F), (G) and (H) Statistical analyses of different other clinical parameters derived from E. Error bars represent mean ± SD of pooled results from n = 6 mice. (I) Spleen cells of EAE mice treated like in A were analyzed 8 or 16 days after EAE induction for their Foxp3^+^ Treg relative frequencies or Ki67^+^ proliferating Foxp3^+^ Treg frequencies. (J) Like in I, but the spleen cells were re-stimulated with 10 μg/ml MOG_35-55_ peptide and then the relative frequencies of Treg expansion were determined after 5 days. (K) Like in I, but splenocytes were re-stimulated with graded concentrations of MOG_35-55_ peptide and after 3 days their supernatants analyzed by ELISA for IL-17A and IFN-γ production. (I-K) Error bars represent mean ± SD of pooled results from n = 6 mice. One Way ANOVA, Dunnett post-test. *p<0.05, **p<0.01, ***p<0.001, ns: not significant.

Since CT^lo^- and cAMP-DCs exerted dominantly tolerogenic functions *in vitro* by inducing Tregs supported by CTLA-2, we tested if the injection of CT^lo^- and cAMP-DCs loaded with the auto-immunogenic peptide MOG would be able to prevent EAE, using the same protocol as we applied before for tolerogenic and semi-mature TNF-stimulated DCs [17,30]. Three *i*.*v*. injections of either DC type were given to each mouse at days -7, -5, -3, before EAE was induced at day 0. While CT^lo^-DCs showed an elevated clinical score at early time points the late disease score and the maximum score remained lower than that of the PBS-treated control group without influencing the day of onset or incidence (Fig 4A to 4D). The clinical score of animals injected with cAMP-DCs appeared both delayed and lower with changes in the day of onset and reduced incidence (Fig 4A to 4D). Control mice injected with CT^lo^- and cAMP-DCs that were not pulsed with MOG peptide as well as mice injected with untreated-DC (immature) did not show significant differences in day onset, in the maximum score or in the incidence of the disease compare with PBS-treated mice, indicating the requirement for MOG peptide loaded on the CT^lo^/cAMP-DC (Fig 4E to 4H).

When the splenic Treg population was analyzed before the onset (d8) and around the peak of disease (d16), increased frequencies of total and proliferating CD4^+^CD25^+^Foxp3^+^ were observed only at d8, but not at d16 in animals injected with CT^lo^- and cAMP-DCs (Fig 4I). Re-stimulation of spleen cells with MOG peptide indicated also a higher frequency of MOG-specific Treg in the CT^lo^- and cAMP-DC treated groups (Fig 4J) but hardly any MOG-specific IL-17A and IFN-γ at day 8 (Fig 4K). This picture dramatically changed at day 16, where those animals protected from EAE by the treated-DC injections showed lower frequencies of Tregs, a complete abrogation of MOG-specific IL-17A production and partially elevated levels of IFN-γ in the spleen (Fig 4J and 4K). Together these data suggest that injections of MOG-loaded CT^lo^- and cAMP-DCs partially protect mice from clinical EAE symptoms. The observed expansion of MOG-specific Foxp3^+^ Tregs before the onset of the disease correlates with the inhibition of MOG-specific Th17 cell development while not suppressing the IFN-γ response.

## Discussion

Besides the accepted central roles of DCs to initiate T cell activation, to instruct polarized T helper cell responses and to control T cell tolerance, the molecular basis underlying these polarizing events are much less clear. Here we found that the CT, a pathogen product that would naturally be secreted by *Vibrio cholerae* bacteria, exerts two types of instructive signals on DCs differentially influencing CD4^+^ T cell polarization. DCs treated under CT^hi^ conditions secrete the Th17-polarizing cytokines IL-1β, IL-6 and IL-23 but also CTLA-2, a molecule that has so far not been identified to be produced by DCs. CTLA-2α expression in retinal pigment epithelial cells has been found to contribute to the immune privilege in the eye by induction of Foxp3^+^ Tregs [39]. Lowering the dose of CT or treating with the intracellular downstream signal mediator cAMP results in a weaker DC maturation characterized by the up-regulation of surface maturation markers and the production of CTLA-2, but lacked the production of Th17 polarizing cytokines. As a result CT^hi^-DCs induced preferentially Th17 responses, while CT^lo^-DCs promoted TGF-β dependent Treg conversion from naive T cells similar to what had been found in the eye.

While the polarization of CD4^+^ T cells into all known Th cell subsets depends on cytokine signals, different groups seem to exist that are linked to each other by counter-regulation. It appears that polarized Th1 and Th2 induction are linked, as well as the induction of Th17 and Foxp3^+^ Tregs. For induction of Th1 immunity the production of IL-12p70 has been defined as a clear Th1 polarizing signal [45], while other data indicate that the signal strength for T cells such as with high doses of antigen also favors Th1 polarization [46–48]. On the other hand, Th2 induction may just require the absence of IL-12p70 or lower doses of antigen [17,49,50] or IL-4 produced by innate cells [51–53]. In contrast, TGF-β is critically involved to promote both Th17 and Foxp3^+^ Tregs, while additional production of IL-1β, IL-6 and IL-23 shift the balance towards Th17 cells and retinoic acid towards Tregs [54]. Since Th17 and Treg generation are linked, it is tempting to speculate whether cholera bacteria may exploit this link by balancing Th17 immunity with Treg tolerance through induction of CTLA-2 production by DCs.

Our microarray data also indicated an induction of Ctla2a and Ctla2b by CT, which could be confirmed by sqPCR. The induction of CTLA-2 was unique to CT and cAMP and not observed for TNF or LPS or as previously shown for two different *Trypanosoma* antigens [17]. Lowering the dose of CT or using its intracellular signaling molecules cAMP for DC treatment abrogated DC cytokine production, whereas the surface marker maturation and the production of CTLA-2 were maintained. While LPS-DCs failed to exert tolerogenic functions in the Treg conversion assays *in vitro*, CT^lo^- and cAMP-DC clearly showed TGF-β induced Treg conversion that was partially dependent on CTLA-2.

While the injection of tolerogenic DCs represents a therapeutic option for clinical application in transplantation and autoimmunity, their stable tolerogenicity is required to avoid secondary maturation and thereby shifting to immunogenic DC phenotypes [55,56]. Our data indicate that tolerogenic DC maturation under CT^lo^ and cAMP conditions may easily switch to an immunogenic DC maturation when CT doses increase. Thus, the identification and application of defined molecular mediators of DC tolerance such as CTLA-2 may be advantageous over the treatment with whole tolerogenic DC. Although a large variety of DC subsets treated in different ways have been tested to achieve suppression or tolerance in animal models, the molecular basis of their tolerogenicity is often much less clear. Here we found that CTLA-2 production by DCs cooperates with TGF-β to convert and expand Tregs. It will be interesting to further investigate tolerogenic functions of CTLA-2 secreting DCs or the CTLA-2 molecule alone for their therapeutic potential as exemplified here in the EAE model.

Along this line cAMP-treated DCs may offer new possibilities to generate human tolerogenic DCs. For this, the interesting question remains if there is a human counterpart for mouse CTLA-2. Although a homologous gene has not been identified in human, one known CTLA-2 binding partner is murine cathepsin L. The murine cathepsin L molecule has a lower protein sequence homology and identity with human cathepsin L as compared to human cathepsin V [57] and both, murine cathepsin L and human cathepsin V, show similar tissue expression pattern and functional activity for invariant chain degradation [58]. Therefore, a functional homologue of murine CTLA-2 may exist in human DCs, but potentially as an interaction partner of cathepsin V.

Previous work suggested that a semi-mature stages of DCs such as achieved with TNF may still allow tolerogenic functions on CD4^+^ T cells when injected repetitively into mice [28]. Key features of this semi-maturation were their upregulation of MHC-II and costimulatory molecules such as CD80, CD86 or CD40 but the failure to secrete cytokines. The phenotype of semi-mature BM-DCs generated *in vitro* is highly similar to steady state migratory DCs that transport self-antigens to the lymph nodes, where they tolerize CD4^+^ T cells by converting them into Foxp3^+^ Tregs [29,59]. Here we found that semi-mature CT^lo^- and cAMP-DCs appear with a partially similar, but also distinct phenotype and CT^hi^-DCs with some similarities in the transcriptional gene signature as compared to semi-mature TNF-DCs. Our previous data suggested that TNF-DCs showed a weak inflammatory gene signature that led to polarization of CD4^+^ T cells into a Th2 response that, upon repetitive stimulation, shifted toward tolerogenic IL-10^+^ Tr1 cells [17,30]. Here we find that CT^hi^-DCs showed a similar inflammatory gene expression profile (Il1b, Il6, Ptgs2 = COX2) and the production of Th17 promoting cytokines IL-1β, IL-6 and IL-23 by ELISA.

Studies with a fusion protein of rCTLA-2α bound to an IgG molecule did not indicate a direct binding of CTLA-2 to other immune cells (unpublished data). For CTLA-2β dimerization formation has been described [33] that may also be required to exert its tolerogenic functions including potential binding to a hypothetical cell surface receptor of the TGF-β family, similar to other TGF-β signaling enhancers such as activin A [60]. These data may point to an interaction of CTLA-2 dimers with TGF-βR family members or promoting conversion of latent TGF-β to active TGF-β to enhance the observed Treg conversion [61,62]. The proteolytic cleavage of latent TGF-β from murine and rat fibroblast cell lines by cathepsin D [63] or human latent TGF-β by cathepsin B [64,65] into its active form has been described before. Human cathepsin L can degrade the single-chain isoform of cathepsin D [66]. It is conceivable that CTLA-2 as a cathepsin L inhibitor may promote TGF-β indirectly via cathepsin D. However, in our experiments Ctsl^-/-^ DCs were able to convert Tregs to a similar extent as observed with WT DCs. It remains to be tested whether CTLA-2 directly interferes with cathepsin D or other molecules to promote latent TGF-β conversion.

CT has been proposed as a vaccine adjuvant. Different routes of administration have been tested to work successfully, including subcutaneous [67], epicutaneous [68] and oral administration [69]. Nasal application in animals with EAE ameliorated their clinical scores [70]. The adjuvant effect of intravenously injected CT was dependent on DCs residing in the marginal zone [71]. However, microbes that are well adapted to their hosts allow a balanced immune response that enables microbial control and host survival, but also counter-regulatory immune evasion mechanisms that prevent complete microbial elimination. The use of microbial substances as vaccine adjuvants may be counteracted by unwanted immune evasion strategies from the respective microbe. Several immune evasion strategies have been described for *Vibrio cholerae*, however not the induction of CTLA-2 [72]. Thus the tolerogenic effects observed by CTLA-2 activity may represent a novel mechanism of immune evasion mediated by CT.

## Conclusion

**Fig 5:**
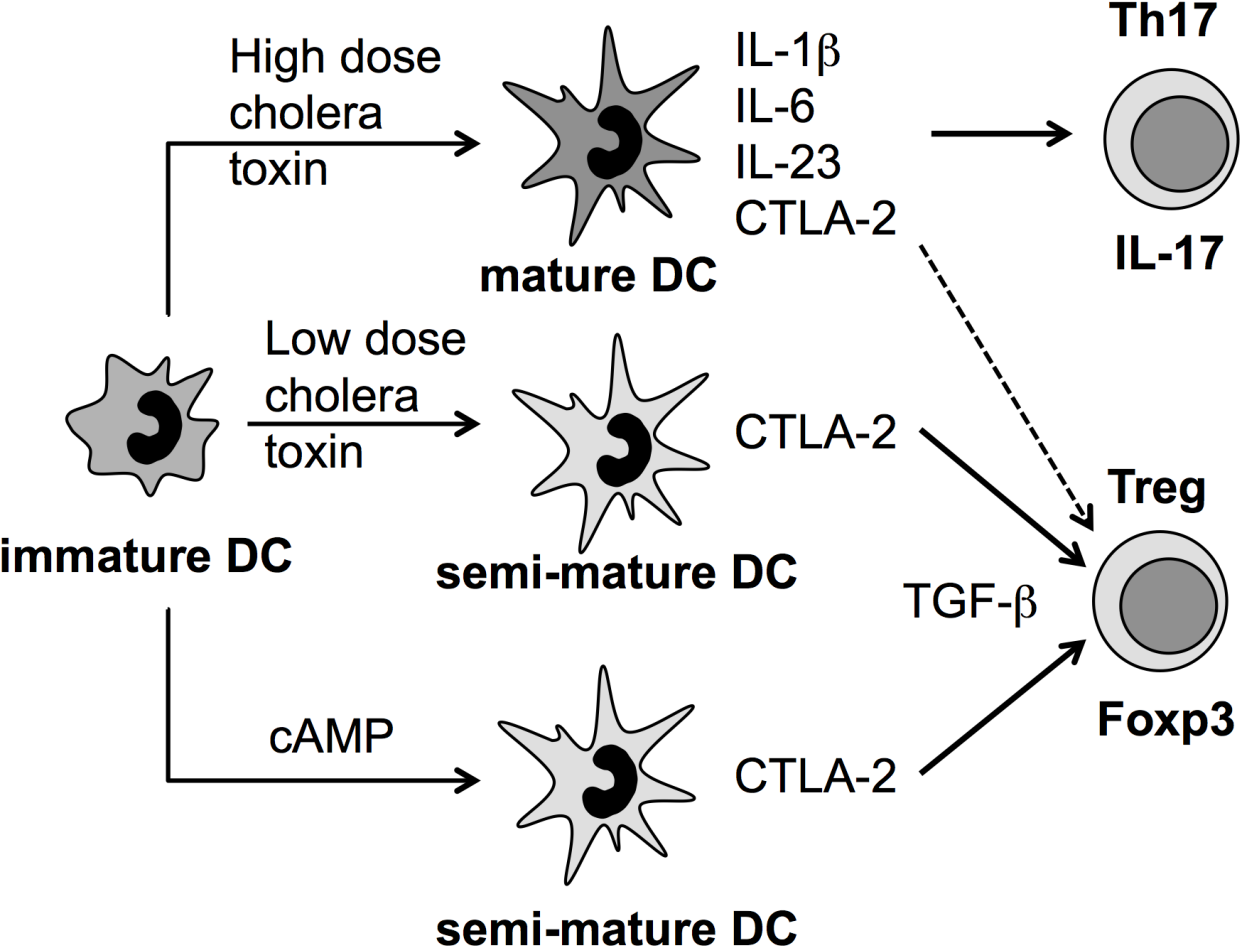
Model on the differential effects of CT and cAMP on DC-directed induction of Th17 or Foxp3^+^ Treg cells. Immature DCs treated with high (CT^hi^) or low (CT^lo^) doses of cholera toxin or its known secondary mediator cAMP induced partial DC maturation measured as upregulation of MHC II- and costimulatory molecules such as CD86. CT^lo^-DCs and cAMP-DCs produced no pro-inflammatory cytokines (semi-mature DCs) but instead the tolerogenic molecule CTLA-2. Under Treg polarizing conditions *in vitro*, using naive TCR-transgenic CD4^+^ T cells, CTLA-2 produced by CT^lo^-DCs and cAMP-DCs supports the TGF-β mediated conversion into Foxp3^+^ Tregs. In contrast, CT^hi^-DCs also released CTLA-2 but additionally the Th17-polarizing cytokines IL-1β, IL-6 and IL-23 (mature DCs). Consequently CT^hi^-DCs mainly generated Th17 cells *in vitro*. *In vivo*, a polyclonal repertoire of T cells triggered by CT^lo^-DCs and cAMP-DCs results in both Th17 and Tregs activation pointing to competing effects at early or late time points in the EAE model.

Here we show that the tolerogenic molecule CTLA-2 can be produced by DCs after stimulation with CT. High doses of CT induce Th17 polarizing cytokines and thereby favoring the polarization into this T cell subset. Low doses of CT or its mediator cAMP induce a DC semi-mature state characterized by the upregulation of surface MHC II and costimulatory molecules in the absence of pro-inflammatory cytokine secretion. In addition and thereby different from the semi-mature TNF-DC phenotype, both treatments also induce the expression of CTLA-2, which cooperates with TGF-β for the conversion of Foxp3^+^ regulatory T cells (Fig 5). Thus, CTLA-2 molecules may represent promising candidates for further studies to treat autoimmune diseases, but also to better understand immune evasion of cholera bacteria.

## Materials and methods

### Animals

The animal experimetns have been permitted by and were controlled through the Regierung von Unterfranken, AZ55.2–2531.01-52/14. C57BL/6 (Charles River), OT-II (kindly provided by F. Carbone, Melbourne), OT-II/*Rag1*^-/-^ mice (RAG1^-/-^ from JAX mice) and cathepsin L^-/-^ (*Ctsl*^*-/-*^) mice [73] were bred under pathogen-free conditions in our facilities. All animal experiments were performed using age- and sex-matched animals. The mice were sacrificed by CO_2_ inhalation according to approved standard operating procedures. For EAE experiments, the animals were immunized or pertussis toxin-injected according to approved standard operating procedures.

### Flow cytometry

Surface staining: performed at 4°C using PBS supplemented with 0.1% BSA and 0.1% sodium azide (FACS buffer). mAbs were purchased from BioLegend, unless otherwise indicated. Pacific Blue-conjugated mAb: anti-CD4 (GK1.5). FITC-conjugated mAbs: anti-MHC-II (M5/114.15.2, BD Biosciences), anti-CD86 (GL1), anti-Vβ5.1 and 5.2 TCR (MR9-4). PE-conjugated mAbs: anti-MHC-II (M5/114.15.2), anti-CD86 (GL1), anti-CD25 (PC61), anti-Vβ5.1 and 5.2 TCR (MR9-4). APC-conjugated mAbs: anti-CD11c (N418), anti-CD25 (PC61). PerCP/Cy5.5-conjugated mAb: anti-CD4 (GK1.5). PE/Cy7- conjugated mAb: anti-CD25 (PC61). Fc receptors were blocked by pre-incubating the cells with 10% of supernatant from 2.4G2 hybridoma (anti-Fc-γ-RII/III; ATCC) prior to staining. Intracellular staining: cells were fixed using 2% formaldehyde, permeabilized with perm buffer (0.5% saponin in FACS buffer) and stained at 4°C using the following Abs in perm buffer: anti-IFN-γ-FITC (XMG1.2), anti-IL-17A-PE (TC11-18H10; BD Biosciences). Nuclear staining of Foxp3 and Ki67: performed using the eBioscience anti-mouse Foxp3 staining set according to the manufacturer’s instructions. Anti-Foxp3-Alexa Fluor 647 (150D) and anti-Ki67-PE or Alexa Fluor 647 (16A8) antibodies were used. Samples were measured in LSRII flow cytometer (BD Biosciences) and data were analyzed with FlowJo software v8.7 (TreeStar).

### Generation of BM-DCs

Murine BM-DCs were generated as previously described [74]. Briefly, bone marrow precursor cells were obtained by perfusion from femur and tibias of C57BL/6 mice and seeded in petri dishes (100 mm, Greiner) at 3×10^6^ cells/dish in 10 ml of complete medium: RPMI 1640 (Sigma) supplemented with 10% heat-inactivated FCS (GIBCO), 100 U/ml penicillin (Sigma), 100 μg/ml streptomycin (Sigma), 2 mM L-glutamine (Sigma) and 50 μM β-mercaptoethanol (Sigma-Aldrich). To induce the differentiation to DCs, the medium was additionally supplemented with 10% of a supernatant obtained from the X-63 cell line transfected with the GM-CSF murine gene [75]. The cells were culture at 37° C, 7% CO_2_. On day 3 of culture, 10 mL of complete RPMI medium supplemented with 10% GM-CSF-containing supernatant was added. On day 6, 10 ml of the medium was removed and fresh medium was added as at day 3. In some cases (EAE), the cells were fed again at day 8.

### CTLA-2 induction in BM-DCs and cAMP inhibition

Day 8 BM-DCs were cultured in complete medium at 1×10^6^ cells/ml in the presence of 10% GM-CSF-containing supernatant without (untreated) or with different stimuli: 0.1 μg/ml cholera toxin (Sigma-Aldrich) as low dose (CT^lo^), 1 μg/ml cholera toxin as high dose (CT^hi^), 100 μM 8-CPT-cAMP (cAMP, Proteinkinase.biz), 0.1 μg/ml LPS (from *E*. *coli*, Sigma-Aldrich) and 500 U/ml TNF-α (Peprotech). The adenylate cyclase inhibitor KH7 (TOCRIS) was used at 100 μM to inhibit cAMP. Total RNA was extracted from DCs using TRIzol® reagent (Invitrogen-Life Technologies) according to the manufacturer’s instructions. cDNA was synthesized using BioScript™ Reverse Transcriptase (Bioline). The relative mRNA levels of both CTLA-2α and β was determined by sqPCR using the following primers: CTLA-2αF, 5’-CTTCAGTGCTGTCTTCCTGCTCAT-3’;

CTLA-2αR, 5’-TTACTCTGGCTGAGCCCTTCCA-3’;
CTLA-2βF, 5’-GGACAACAAAGTTCTGGTTTCTATCTG-3’;
CTLA-2βR, 5’-AACTGTTACTCTGGCTGAGCCCTT-3’;
β-actinF, 5’-CCTAGGCACCAGGGTGTGAT-3’;

β-actinR, 5’CTCTTTGATGTCACGCACGATTTC-3’. The PCR products were separated by electrophoresis using a 2% agarose gel. The bands were visualized by staining with ethidium bromide. Amplicons were quantified by densitometry using ImageJ v1.48 software [76] upon normalizing using a β-actin control.

For microarray analysis bone marrow precursor cells were depleted of T and B cells using anti-CD90.2 and anti-CD19 magnetic beads (Miltenyi Biotec), respectively. Day 8 BM-DCs were stimulated as mentioned above and total RNA was extracted. Samples were prepared and microarray analysis performed as previously described [17]. RNA integrity and comparability between samples was tested using a BioAnalyzer (Agilent). RNA integrity numbers were between 9,8 and 10.

### Cytokine production by BMDC

The supernatants of matured BM-DCs were collected and centrifuged at 10,000×g for 10 min prior to measurement. IL-1β, IL-6 (both BD Biosciences) and IL-23 (eBioscience) production were determined by ELISA. Absorbance was detected using the SpectraMax Plus 384 Microplate Reader (Molecular Devices).

### *In vitro* Th17 and Th1 differentiation

Single cell suspension from lymph nodes (LNs) of OT-II mice were obtained by gently mashing and passing through a 70 μm cell strainer. 1×10^5^ bulk cells were co-cultured in round bottom 96-well plates together with 2×10^4^ day 8 BM-DCs treated for 4 h with different stimuli as indicated, in the presence of 100 ng/ml OVA peptide_327-339_ (Charité-Berlin), 2 ng/ml porcine TGF-β1 (R&D Systems), 20 ng/ml rmIL-6 (ImmunoTools) for Th17 differentiation or 100 ng/ml OVA peptide_327-339_, 5 ng/ml IL-12 (Peprotech) in the case of Th1 polarization. After 5 days at 37°C and 7% CO_2_ in 200 μl of IMDM medium supplemented as RPMI, the cells were harvested, counted and re-stimulated at 1×10^6^/ml in complete IMDM with 30 ng/ml PMA (Sigma-Aldrich) and 1 μg/ml Ionomycin (Sigma-Aldrich). After 1 h of stimulation 5 μg/ml Brefeldin (Sigma-Aldrich) and 2 μM Monensin (Sigma) were added without washing and further incubated for 5 hours. IL-17A and IFN-γ production were measured by flow cytometry staining as mentioned above.

### *In vitro* Treg conversion assays

Naïve CD4^+^CD25^-^ T cells were obtained from spleen and LN of OT-II mice. After erythrocyte lysis, total CD4^+^ cells were negatively selected using the CD4^+^ EasySep kit (StemCell Technologies) according to manufacturer’s instructions. Then, CD4^+^CD25^-^ cells were acquired after magnetic isolation of CD4^+^ cells using the CD25 microBeads kit (Miltenyi). 2×10^4^ naïve T cells were co-cultured in round bottom 96-well plates together with day 8 BM-DCs treated for 4 h with different stimuli as described above, in the presence of 100 ng/ml OVA peptide_327-339_ and 2 ng/ml porcine TGF-β1. The cells were cultured in 200 μl of complete RPMI medium at 37° C, 7% CO_2_. The conversion of naïve T cells towards iTreg was analyzed after 5 days by Foxp3 staining.

For polyclonal Treg conversion assay, naïve CD4^+^CD25^-^ T cells were negatively selected as before from spleen and lymph nodes of C57BL/6 mice. 3–5×10^5^ naïve T cells were cultured in 24-well plates pre-coated overnight at 4° C with 5 μg/ml of anti-CD3 antibody (clone 145.2C11) in the presence of 1 ng/ml porcine TGF-β1 and 500 U/ml rhIL-2 (Novartis) and with or without 10 μg/ml of recombinant CTLA-2α. The cells were cultured in 1 ml of complete RPMI medium at 37° C, 5% CO_2_. The conversion of naïve T cells towards iTreg was analyzed after 3 days by Foxp3 staining.

### Knockdown of CTLA-2 with small interfering RNA (siRNA)

CTLA-2α and β expression in day 8 stimulated BM-DCs was blocked using four different duplex of siRNAs (customer made, Invitrogen-Life Technologies) targeting the CTLA-2 coding sequence. siRNAs were delivered into BM-DCs as previously described [77] with some modifications: BM-DCs (4×10^6^) and siRNAs (final concentration 5 μM) were prepared each one in 100 μl of serum- and phenol red-free Opti-MEM® medium (Gibco). Then, BM-DCs were electroporated with the siRNAs in 4-mm electroporation cuvettes (Peqlab) at room temperature using the Gene Pulser Xcell (Bio-Rad). Pulse conditions were 400 V, 6 ms, 150 μF. Immediately after the pulse, an equivalent volume of complete RPMI medium was added and the electroporated cells were allow to stand for 1 h at 37° C, 7% CO_2_. The cells were washed with complete RPMI medium and treated for 4 h with diverse stimuli as indicated prior the co-culture with OT-II T cells for Th17, Th1 or Treg differentiation.

The sequences of siRNAs used to target CTLA-2α and β coding sequences at different sites were used alone or mixed (maintaining a final concentration of 5 μM) with similar results. A mouse scramble siRNA was used as a control (Ambion-Life Technologies). The efficiencies of knockdown were calculated by setting the scramble control as 100% and revealed 66±17.7 for CTLA-2a and 76±10.7 for CTLA-2b. (as % decrease). The sequences of siRNAs used were the following:

127s, 5’-AUAGUCUGCAUUGUGUGCCTT-3’;
127a, 5’-GGCACACAAUGCAGACUAUTT-3’;
202s, 5’-GGAUAAUGAGUGGAAAGAATT-3’;
202a, 5’- UUCUUUCCACUCAUUAUCCTT-3’;
213s, 5’-UUCGUCUUCCAUUCUUUCCTT-3’,
213a, 5’-GGAAAGAAUGGAAGACGAATT-3’;
495s, 5’-GCUUGACUGGUAACAAUAUTT-3’,
495a, 5’-AUAUUGUUACCAGUCAAGCTT-3’.

### Production of recombinant CTLA-2α

Total RNA was extracted from CT^hi^-treated DCs and first-strand synthesis of cDNA was performed using Moloney Murine Leukemia Virus Reverse Transcriptase, RNase H Minus, Point Mutant polymerase (Promega) according to manufacturer’s instructions. Phusion High-Fidelity polymerase (New England biolabs) was used for second strand cDNA synthesis and PCR amplification. The primers used were:

CTLA-2a-a, 5’-TTACCAGTCAAGCCACAGCTGTTACTCTGGC-3’;
NdeCTLA-2a-alphas, 5’-AACCATATGATGGTTTCTATCTGTGAACAGAAGCTGC-3’.

The PCR product was gel purified using GenElute Gel Extraction Kit (Sigma-Aldrich) according to the manufacturer’s instruction. The amplicon was cloned into the vector pSC-B-amp/kan (Stratagene) following manufacturer’s instructions and verified by nucleotide sequencing. The CTLA-2α encoding sequences were excised and inserted in the mammalian expression vector pcDNA3.1+ (Invitrogen) and into a bacterial expression vector following an N-terminal His tag (BioLegend). The resulting plasmids were sequenced.

Bacterial expression of recombinant CTLA-2α was induced with 1 mM isopropyl-β-D-thiogalactopyranoside (IPTG) when OD_600_ had reached 0.6 and expression was continued for ~3 hours at 37°C. Cell were lysed in 20 mM Tris (pH 7.5) and 300 mM NaCl. His-tagged CTLA-2α was purified by affinity chromatography using HisTrap FF columns (GE Healthcare) and eluted in, with 300 mM imidazole. The eluted fractions were dialyzed overnight at 4°C against a buffer composed of 20 mM Tris and 100 mM NaCl. The His-tag was removed by factor X cleavage and the CTLA-2α was purified by reverse His-tag purification. The protein was then further purified by Superdex 200 chromatography in PBS buffer. Endotoxin removal was performed using Cellufine ET-clean S (JNC Corporation) following manufacture's instruction.

### Western blot of cathepsin L

Day 8 BM-DCs were treated for 10 h with different stimuli as indicated and the presence of CathL was evaluated by Western blot (SDS-PAGE). The cells were washed with PBS and resuspended in sodium acetate buffer (100 mM sodium acetate, 1 mM EDTA, 0.05% Brij35) at 10^7^ cells/ml. Cell lysates were prepared by freeze-thaw cycles (liquid nitrogen-37° C, three times) and protein concentration was determined using a microplate bicinchoninic acid (BCA) protein assay kit (Thermo Scientific). 10 μg of protein were loaded per lane, separated by SDS-PAGE (12% polyacrylamide gels) and transferred to PVDF membranes. The membranes were blocked overnight at 4° C in 10% non-fat milk and for CathL detection a biotinylated anti-mCathL (R&D Systems) was used followed by incubation with HRP-conjugated streptavidin (Thermo Scientific). The membranes were developed using chemoluminescent HRP substrate (Millipore) and visualized using the FluorChem Q imager (Alpha Innotech). As loading control, the membranes were stripped by incubation with buffer containing 62.5 mM Tris HCl pH 6.7, 2% SDS and 0.8% (v/v) β-mercaptoethanol for 20 min at 70° C. The membranes were then washed with PBS plus 0.1% tween buffer, and blocked overnight at 4° C. β-actin was detected using anti-m-β-Actin (Sigma-Aldrich) as the primary antibody followed by incubation with biotinylated goat anti-mIgG1 (Abcam) and HRP-streptavidin. The membranes were developed and visualized as described above. The images were analyzed by densitometry using ImageJ v1.48 software [76] normalizing regarding β-actin control.

### Experimental autoimmune encephalomyelitis (EAE)

Days 6, 8 and 10-BM-DCs were treated for 4 h with CT^lo^ and cAMP in the presence (or not) of 40 μg/ml of MOG_35-55_ peptide (MEVGWYRSPFSRVVHLYRNGK; Charité-Berlin). 2×10^6^ matured-DCs were injected intravenously into age- and sex-matched C57BL/6 mice at days -7, -5 and -3 before the induction of EAE. Control mice received no DC treatment. The disease was induced as described before [30] with some modifications. 20 μg of MOG_35-55_ peptide were injected subcutaneously in the rear flank in 50 μl of complete Freund’s adjuvant (Sigma-Aldrich) enriched with 10 mg/ml *M*. *tuberculosis* (H37 RA, Difco/BD). In addition, 100 ng/mouse of Pertussis toxin (List Biological Laboratories) were injected *i*.*p*. at days 0 and 2 of EAE induction. Mice were monitored daily for clinical disease symptoms according to the following scale: 0 = no disease; 1 = tail weakness; 2 = full tail paralysis; 3 = hind limp paralysis; 4 = hind limp and back paralysis; 5 = fore limp paralysis or moribund or death.

The frequency and activation state of Treg were measured in freshly isolated and in re-stimulated splenocytes of mice after 8 and 16 days of EAE induction. In the latter case, the cells were cultured for 5 days at 1×10^6^/ml in BioWhittaker™HL-1™ (Lonza) free serum medium in the presence of 10 μg/ml MOG_35-55_ peptide. Additionally, splenocytes were re-stimulated for 3 days at 2×10^6^/ml in BioWhittaker™HL-1™ with graded concentration of MOG_35-55_ peptide to measure IL-17 and IFN-γ production in the supernatants by ELISA (R&D Systems and BD Biosciences, respectively). The supernatants were collected and centrifuged at 10,000×g for 10 min prior to measurement.

### Statistical analysis

Data are presented as the mean ± SD, except for the EAE score, where SEM is displayed. The data were analyzed utilizing Student t-test or One-way Anova using Dunnett post-test using Graph Pad Prism™ software. Significance level was defined as *p<0.05, **p<0.01, *** p<0.001.

## Abbreviations

CT: cholera toxin
DC: dendritic cell
iTreg: CD4^+^CD25^+^Foxp3^+^ T cells
Ctsl: cathepsin L
MS: multiple sclerosis
EAE: experimental autoimmune encephalomyelitis
